# Smart Skin: Vision-Based Soft Pressure Sensing System for In-Home Hand Rehabilitation

**DOI:** 10.1089/soro.2020.0083

**Published:** 2022-06-08

**Authors:** Yuanfeng Han, Aadith Varadarajan, Taekyoung Kim, Gang Zheng, Kris Kitani, Aisling Kelliher, Thanassis Rikakis, Yong-Lae Park

**Affiliations:** ^1^ Department of Mechanical Engineering, Johns Hopkins University, Baltimore, Maryland, USA.; ^2^ Robotics Institute, Carnegie Mellon University, Pittsburgh, Pennsylvania, USA.; ^3^ Department of Mechanical Engineering, Institute of Advanced Machines and Design, Institute of Engineering Research, Seoul National University, Seoul, Korea.; ^4^ Department of Computer Science, Virginia Polytechnic Institute and State University, Blacksburg, Virginia, USA.; ^5^ Department of Bioengineering, Virginia Polytechnic Institute and State University, Blacksburg, Virginia, USA.

**Keywords:** soft robotics, soft sensors, force sensors, Smart Skin, hand rehabilitation, computer vision, image processing

## Abstract

We introduce a novel in-home hand rehabilitation system for monitoring hand motions and assessing grip forces of stroke patients. The overall system is composed of a sensing device and a computer vision system. The sensing device is a lightweight cylindrical object for easy grip and manipulation, which is covered by a passive sensing layer called “Smart Skin.” The Smart Skin is fabricated using soft silicone elastomer, which contains embedded microchannels partially filled with colored fluid. When the Smart Skin is compressed by grip forces, the colored fluid rises and fills in the top surface display area. Then, the computer vision system captures the image of the display area through a red–green–blue camera, detects the length change of the liquid through image processing, and eventually maps the liquid length to the calibrated force for estimating the gripping force. The passive sensing mechanism of the proposed Smart Skin device works in conjunction with a single camera setup, making the system simple and easy to use, while also requiring minimum maintenance effort. Our system, on one hand, aims to support home-based rehabilitation therapy with minimal or no supervision by recording the training process and the force data, which can be automatically conveyed to physical therapists. In contrast, the therapists can also remotely instruct the patients with their training prescriptions through online videos. This study first describes the design, fabrication, and calibration of the Smart Skin, and the algorithm for image processing, and then presents experimental results from the integrated system. The Smart Skin prototype shows a relatively linear relationship between the applied force and the length change of the liquid in the range of 0–35 N. The computer vision system shows the estimation error <4% and a relatively high stability in estimation under different hand motions.

## Introduction

Stroke is one of the most common neurological disorders over the world caused by either blockage of a blood vessel carrying blood to the brain—*ischemic stroke*—or leakage of blood into the brain from a broken blood vessel—*hemorrhagic stroke*.^1^ According to the American Heart Association, someone in the United States has a stroke every 40 seconds,^2^ and there are estimated seven million stroke survivors in the United States.^3^ After initial hospitalization, ∼80% of stroke survivors return to the community, needing assistance from family caregivers for activities of daily living.^4^ Many of them experience hemiparesis, weakness of one side of the body, including at least one hand, which causes difficulty in carrying out everyday activities, such as eating, dressing, and personal hygiene, due to loss of dexterity in manipulating simple daily life objects.

Recent studies show that long-term rehabilitation in the clinic can be effective in supporting stroke recovery.^5–7^ However, the scalability and sustainability of this approach over time are limited due to issues of cost,^8^ availability of experts, number of facilities, and transportation challenges.^9^ Recent studies show that community in-home rehabilitation is effective in improving the functional and the psychosocial recoveries of patients after stroke,^10^ and remote therapy can be acceptable for patients with well-designed procedures.^11^ As a result, in-home rehabilitation is now being considered as a viable effective complement, or even alternative, to traditional clinic or hospital based therapy methods.^12^

Different types of rehabilitation systems with varying potential for home-based therapy have been proposed for long-term training. Several systems address walking challenges for stroke survivors, including robotic rehabilitation exoskeleton machines,^13^ and active knee orthosis for correcting hemiparetic walking gaits.^14,15^ In addition, soft wearable robotic devices have also been developed for lower body^16,17^ and hand^18,19^ rehabilitation of poststroke hemiplegic patients.^20^ Robotic and game-based systems have been proposed to assist with upper limb and hand training, such as a home-based Computer Assisted Arm Rehabilitation robotic device used for upper limb exercises for poststroke patients^21^ and virtual rehabilitation systems using off-the-shelf game gloves for poststroke hand training.^22^

However, the majority of these systems require relatively complicated setup and initialization procedures with multiple mechanical parts, which make it difficult for stroke patients and their caregivers to effectively and efficiently use them in the home.

In general, rehabilitation therapy for stroke patients consists of repetitive movement tasks, such as reaching, grasping, and manipulating objects under the supervision of a physical therapist.^23,24^ The performance of the patient is evaluated by the therapist, who provides feedback to the patient ranging from prescriptive to suggestive comments and statements. One important measure that is difficult to evaluate during grasping tasks is the applied force (or pressure) on the object by the patient's hand. To detect this force, sensors typically need to be embedded in, or attached to, the training object.

However, traditional pressure sensors are generally made of rigid electronic components, and object sensing areas are subsequently limited by the size and the number of sensors. This makes it difficult to integrate sensors in a training object with different shapes and sizes, while also posing difficulties in covering the entire grasping area. Furthermore, some sensors are uncomfortable for direct contact with human hands.

**FIG. 1.**
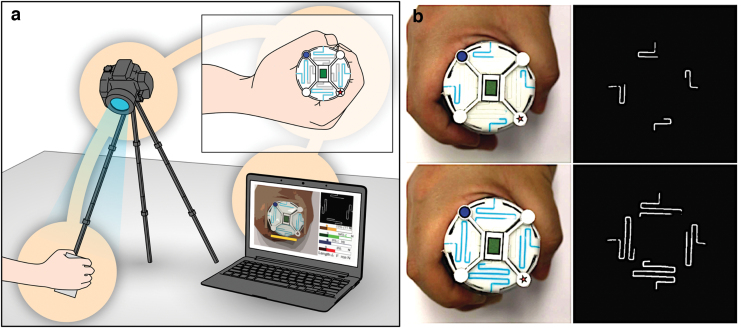
**(a)** Overall concept of the Smart Skin system. **(b)** Smart Skin hand rehabilitation device with different grip forces (*left*) and their processed images for estimating the forces by computer vision (*right*). Color images are available online.

Therefore, we propose implementation of a soft sensor technology for grasp force sensing in training objects aimed at refining human–device interactions and increasing the degree of comfort. We developed an in-home hand rehabilitation and monitoring system (Fig. 1) for stroke survivors. The system uses a novel vision-based soft sensor called “Smart Skin” to detect and record grip force data during rehabilitation activities.

On one hand, the system aims to support home-based rehabilitation therapy with minimal or no supervision, in the way that the recorded training video and force information can be saved and delivered to the physical therapists. On the other hand, the physical therapists can provide instruction for training after watching the Supplementary Video S1 of the training progress from the patient. Moreover, the design and form factor of the system help to significantly reduce the cost and physical footprint of the approach compared to other clinic- or home-based systems. In addition, the Smart Skin device requires no electronic components and consequently increases the degree of comfort of the users.

Soft sensors have been investigated for decades using different types of polymer materials with embedded sensing mechanisms. One of the most traditional approaches is to mix conductive particles in micro/nano scales with elastic silicone rubbers.^25–29^ When the matrix polymer deforms, the distances between the embedded particles change, consequently changing the electrical resistance or the capacitance of the entire material. Although the mechanism and fabrication are simple, these sensors tend to show relatively high hysteresis, as well as to lose their high compliance and elasticity with an increased concentration of the conductive particles.^30^

Another approach, a more recent development, is to use highly stretchable rubber materials with embedded microchannels filled with conductive liquids, such as room-temperature liquid metals^31–33^ or ionic liquids.^34,35^ When deformed, the conductive microfluidic channels change their geometries, resulting in changes of their electrical resistances, which allow for sensing of different deformation modes, such as strains,^36–38^ forces,^39–43^ or curvatures.^44,45^ They can be also designed to detect capacitance changes depending on applications.^46^ Although this method provides more reliable sensor signals than conductive polymers, it usually involves complicated manufacturing processes.

Optical sensing is another method of soft sensing. Deformation can be detected based on the reflection wavelengths or the optical power losses by optical sensors,^47–49^ fiber optics,^49,50^ or optical waveguides^51–54^ embedded in a soft structure.

However, all the above soft sensors require electronic components to acquire, collect, and transfer the sensor data, which means that the users (i.e., stroke patients) need to be actively involved with continuous maintenance of the device, such as changing or recharging batteries and turning on/off the device, while working on their own rehabilitation. Although these tasks might be easy and trivial to healthy people, they could be an additional burden to people with disability to whom most of the daily activities are already heavy burdens both physically and mentally. Furthermore, if the device contains electronics, there is always a possibility of failures.

Therefore, a novel approach that utilizes a microfluidic soft sensing mechanism combined with a computer vision technique is implemented in our rehabilitation system. We embed a colored fluid in microchannels in a transparent soft polymer layer to detect the magnitudes and the locations of the contact forces. Although microfluidic displays have been proposed for color change of soft robots^55^ and tactile sensing,^56^ the displays were made in a two-dimensional plane and required external sources of fluid.

In our system, when the user grips the three-dimensional (3D) Smart Skin object, a training object covered by the Smart Skin, the colored liquid inside the microchannels is pushed up by the hand pressure and fills the microchannels on the top surface of the object resulting in a color change of the top surface display area. During this procedure, the visual representation of the top area is captured by a camera, processed by image processing, and mapped with the gripping force. When the user releases the object, the liquid flows back to its original location in the microchannels, returning the top surface area to its original color state. In this way, we would like to minimize the number of devices that the user needed to take care of as much as possible. We eventually imagine a system the user can work on the daily in-home rehabilitation program without worrying about checking the powers and maintenance of multiple devices.

## Design

The Smart Skin uses a simple sensing principle. When the external grip forces are applied to the device, the microchannels on the Smart Skin deform, and the colored liquid is pushed up to the display area. Then, a computer vision system recognizes and processes the images of the top area for estimating the length changes of the colored microchannels (Fig. 1, right). Finally, the detected channel lengths are mapped to the precalibrated force data for estimating the grip forces.

### Smart skin

**FIG. 2.**
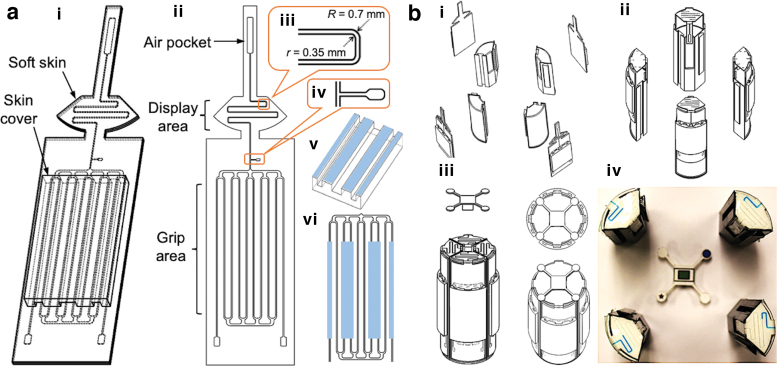
**(a)** Smart Skin design: (i) and (ii) Smart Skin design. (iii) Magnified view of the microchannel in the display area. (iv) Lubricant injection port. (v) Skin cover design showing protrusions along the length of the microchannels. (vi) Areas to be compressed by the skin cover (Six of 10 channels covered by the *blue boxes*). **(b)** Smart Object design: (i) 3D printed four-piece cylinders and Smart Skins. (ii) Smart Skin attachment. (iii) Assembled cylinder and reference cap. (iv) Smart Object prototype with Smart Skin before final assembly. 3D, three-dimensional. Color images are available online.

The Smart Skin is composed of a soft skin and a skin cover (Fig. 2a-i). The soft skin is 1.5 mm thick and can be divided into two sections: the display and the grip areas (Fig. 2a-ii). The microchannels in both areas are originally filled with air and colored liquid, respectively. The grip area channels are all connected by the two horizontal channels at the top and the bottom, which facilitate efficient shifting of the colored liquid from the grip area to the display area when an external force is applied.

The microchannel in the display area (Fig. 2a-iii) has a smaller cross-section (0.35 mm × 0.35 mm) than the microchannels in the grip area. The smaller cross-section reduces the radius difference between the outer and the inner corners and helps prevent the liquid from adhering to the channel walls when the liquid retreats. The walls of the display channels are coated with a lubricant for smooth flow of the colored liquid and to minimize its residues. The lubricant oil is injected through a small port between the grip and the display areas (Fig. 2a-iv) and flushed through the long air pocket at the top of the skin, which is also used for holding air when sealed.

When the colored liquid is pushed up to the display area by external contact forces, the air captured in the display channel moves up and is temporarily compressed in the air pocket. When the external pressure is removed, the compressed air in the pocket pushes the liquid back to its original location. A skin cover (Fig. 2a-v) is used to distribute the grip force uniformly to the pressure areas (light blue areas in Fig. 2a-vi) in the skin. The skin cover is thus made of a stiffer material than the skin material and contains multiple longitudinal protrusions to apply uniform pressure to the skin.

**FIG. 3.**
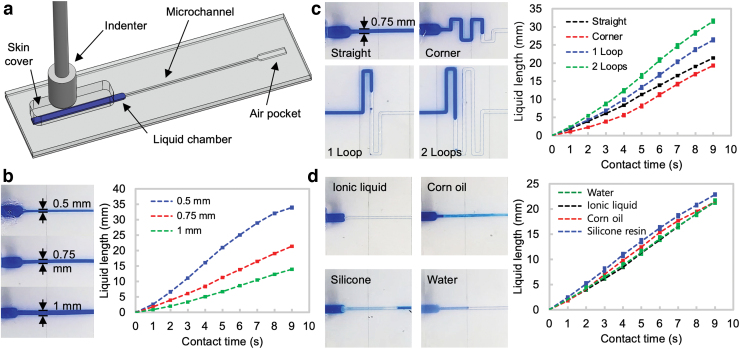
**(a)** Test setup for microchannel characterization. Liquid movement sensitivities for different **(b)** channel widths, **(c)** channel geometries, and **(d)** liquid viscosities—ionic liquid: 97, Corn oil: 60, silicone resin: 1000, and water: 0.9 (unit: cP at 25°C). The *left* four photos in **(d)** show the microchannels after retracting the liquids. Ionic liquid shows clean channel leaving no residues. Color images are available online.

There are various factors that affect the sensitivities of the liquid movement. We tested different microchannel geometries and liquid viscosities to check the sensitivities. Figure 3a shows the setup for testing. A single microchannel with a liquid chamber and an air pocket was prepared, and the chamber was initially filled with colored liquid.

When the skin was compressed by applying a force at a constant speed to the skin cover with an indenter, the liquid moved to the microchannel and its length was measured with contact time. The smaller channel showed the higher sensitivity (Fig. 3b). A meander channel showed a lower sensitivity than a straight channel even though the lengths of the two channels were the same (Fig. 3c, top). This is because the corners of the meander channel slow down the movement of the fluid. In meander channels, the channel with more loops showed a higher sensitivity (Fig. 3c, bottom), since it had longer channel filled with air. When liquids with different viscosities were tested, more viscous liquid showed a slightly higher sensitivity. However, it left more residues when the liquid retracted, as shown in the four photos in Figure 3d. Additional test result can be found in the Supplementary Data with Supplementary Figures S1 and S2.

### Smart object

The Smart Object is the body of the sensing device to which the Smart Skin is attached. We designed the smart object based on the following criteria: (1) The object is required to have a flat area to display the color change of the microchannels, which reduces the challenge for image capturing and processing. (2) The shape of the object should be one of the forms commonly used in clinical rehabilitation training and assessment. (3) The size and the weight of the object should take comfortability and portability into account.

Considering that a cylindrical object is commonly used for hand stroke rehabilitation exercise and grip force assessment,^57,58^ and its top flat surface can be used for displaying the colored fluid, we chose to use a cylindrical shape for the object. The diameter of the Smart Object was designed to be 50 mm (56 mm with the Smart Skin on it), which aimed to maximize the contact area between the human hand and the cylindrical object according to the result on investigation on cylindrical handles.^59^ The weight of the Smart Object together with the Smart Skin is only 185 g, which is significantly lighter than the suggested maximum weight of 450 g for hand rehabilitation devices.^60^ Compared to other hand rehabilitation devices, which considered portability as one of their design factors,^61–64^ our Smart Object is relatively small in size and low in weight, showing high portability for daily usage.

The Smart Object is composed of three major components: a 3D printed cylinder with four sub-pieces, Smart Skin on the cylinder, and a vision reference cap. The four sub-pieces easily lock themselves when assembled. For assembly, a Smart Skin is attached to each sub-piece of the cylinder (Fig. 2b-i), and the four sub-pieces with the Smart Skins are assembled together (Fig. 2b-ii) followed by insertion of the reference cap at the top (Fig. 2b-iii). The cap has unique markers that help identification of the four identical skins when recognized by the computer vision system.

### Material selection

The material for the Smart Skin was selected based on the physical robustness with multicycle uses, low air permeability, as well as the nontoxicity to the user. The soft skin and the skin cover are made of hybrid elastomers of silicone (Ecoflex Series, Smooth-On) and polydimethylsiloxane (PDMS) (SYLGARD 184, Dow Corning), both of which are certified to be safe for interaction with human skin. They also show high shape-recovery rates after being deformed multiple times.

The Smart Skin is made of a mixture of two silicone elastomers: Ecoflex 00-50 and Ecoflex Gel. The Ecoflex 00-50 keeps the skin elastic, while the Ecoflex Gel prevents the liquid in the microchannel from evaporating. Low air permeability of the skin material is critical for the Smart Skin sensor, since reduction of the air pressure in the channel can decrease the reverse flow speed when the colored fluid moves back to its original level. In addition, the elasticity of the material helps the microchannel recover its original shape quickly after deformation. As we experimentally tested the increased mixing ratio between Ecoflex 00-50 and Ecoflex Gel from 1:1, 2:1 till 10:1, we discovered that the mixing ratio of 5:1 showed a good performance in sealing the air, at the same time maximizing the elasticity of the material.

The skin cover is made of a mixture of 80% SYLGARD 184 (PDMS) and 20% Ecoflex Gel in weight, which was also determined through experimental testing. As we gradually increased the mixing ratio between Ecoflex Gel and PDMS, we discovered that the ratio of 1:4 allowed for enough flexibility for the material to conform to the curved surface of the Smart Object while maintaining good enough stiffness.

The synthesized material of the Smart Skin is transparent, and the liquid in the microchannel is clearly visible through the top layer. The bottom layer of the soft skin is dyed in white for a higher contrast with the color of the liquid. For the colored liquid, an ionic liquid (1-ethyl-3-methylimidazolium ethyl sulfate; Sigma-Aldrich) is selected and dyed in blue, since it easily dissolves pigment and is also chemically stable. In addition, the high surface tension of the ionic liquid (72 mN/m at 20°C) helps minimizing the effect of adhesion to microchannel walls while flowing.

#### Material characterization for smart skin

Since no data sheets are available for our custom skin material, the material was experimentally characterized to evaluate its elastic modulus for the analytical models of the Smart Skin. Although a traditional hyperelastic material usually has a nonlinear relationship between stress and strain, we used a linear regression to approximate the elastic modulus of the material as a constant and applied it into the analytical models, since the strain–stress curve was not too far away from linear behavior and the model was based on the linear fracture mechanics under an assumption of the material with a constant elastic modulus. A similar method has been used to model the deformation of the microchannels for a hyperelastic material in the previous work.^32^

A motorized material test-stand (ESM301, Mark-10) and a single-axis load cell (STL-50, AmCells) were used for a stress–strain test. A rectangular skin sample of 30 mm (width) × 60 mm (height) × 20 mm (thickness) was fixed in the test stand (Supplementary Fig. S3a, left) and gradually stretched (Supplementary Fig. S3b, right) up to 40% strain. During testing, the tensile stress and the strain were recorded. The elastic modulus *E* can be found as

$$E=\frac{\sigma }{\delta }=\frac{F∕A}{\Delta L∕L_{0}}$$

where σ and δ are the stress and the strain, respectively, *F* and *A* are the force and the area of stress, respectively, and ΔL and *L*_0_ are the length change and the original length of the sample, respectively. Three identical experiments were conducted, and the data were fitted by linear regression to find the estimated *E*, the slope of the fitted line. The experimental elastic modulus of the skin material was 64.4 kPa.

## Fabrication

**FIG. 4.**
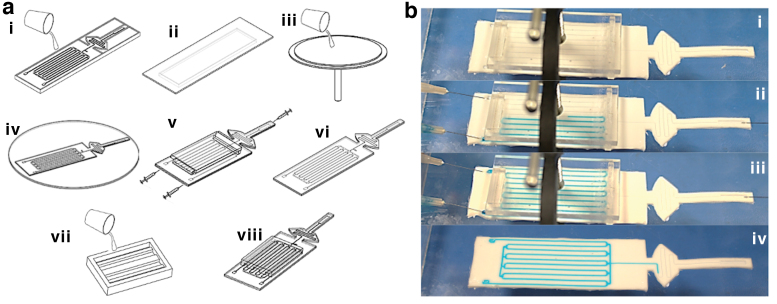
**(a)** Fabrication process: (i) Prepare mold and pour liquid silicone. (ii) Place an acrylic plate on the *top* of the mold and cure. (iii) Spin-coat flat liquid silicon layer and partially cure. (iv) Bond the cured layer in (i) to the flat partially cured layer and cure. (v) Inject colored liquid into the microchannels and seal the pinholes with polymer glue. (vi) Complete skin. (vii) Prepare mold and pour liquid silicone for the skin cover and cure in the oven. (viii) Attach the skin cover on the soft skin. **(b)** Liquid injection process: (i) Block horizontal microchannels with indenter and clamp. (ii) Inject colored liquid making a zig-zag pattern. (iii) Complete injection. (iv) Remove indenter to fill horizontal channels. Color images are available online.

The fabrication process for the Smart Skin can be divided into five steps: base layer casting, top layer casting, layer bonding, liquid injection, and skin cover lamination, as shown in Figure 4a.

The first step is to fabricate a base layer of the skin by molding and casting. A 3D printed plastic mold is prepared, and liquid silicone (80% of Ecoflex 00-50 and 20% of Ecoflex Gel in weight) is poured in the mold (Fig. 4a-i). The uncured silicone is pressed with a flat acrylic piece sprayed with mold release (Ease Release 200, Smooth-On) for squeezing out excessive material and making the layer flat (Fig. 4a-ii). The silicone in the mold is cured at 60°C in an oven for 20 min and removed from the mold.

The second step is to fabricate a top layer of the skin using spin coating. A drop of the same liquid silicone is poured on a flat acrylic substrate and spun at 600 rpm for 1 min in a spin coater. The coated silicone layer is cured with the substrate at 60°C for about 15 min and cooled down to room temperature. When the layer is cured, another layer is spun on top at 2000 rpm for 45 s (Fig. 4a-iii). The new layer is partially cured at 60°C for 30 s.

The third step is to bond the base layer to the spin-coated top layer. The cured base layer is carefully laminated on the partially cured top layer. The bonded skin is cured at 60°C for 5 min (Fig. 4a-iv). While curing, the partially cured silicone on the top layer makes cross-links with both the top and the base layers as a bonding agent. After curing, the skin is removed from the acrylic substrate.

The fourth step is to inject colored liquid into the microchannels. Since the grip area of the skin has multiple microchannels connected together, it is difficult to fill all the microchannels with a single injection. Before injection, the top and bottom horizontal channels are compressed and blocked by a rigid indenter (Fig. 4a-v) so that the microchannels form a single zig-zag line. The colored liquid is injected from one port to the other port to fill the zig-zag channel. After filling the channels in the grip area, mineral oil (Hydrobrite 380 PO, Sonneborn) is injected through the middle port and flushed through the display microchannel for lubrication. The indenter is then removed to fill the compressed channels in the grip area (Fig. 4a-vi). The level of the colored liquid is controlled by the air pressure at the top pocket. The injection holes are sealed by silicone glue. The actual injection process is shown in Figure 4b.

The final step is to attach the skin cover to the soft skin. Similar to the first step, liquid silicone (80% of SYLGARD 184 and 20% of Ecoflex Gel in weight) is poured in a 3D printed mold and cured at 60°C for 2 h (Fig. 4a-vii). When it cures, it is removed from the mold and dipped into thin uncured silicone. Finally, the skin cover is bonded to the skin and cured at room temperature for 30 min (Fig. 4a-viii).

## Characterization

### Force response

**FIG. 5.**
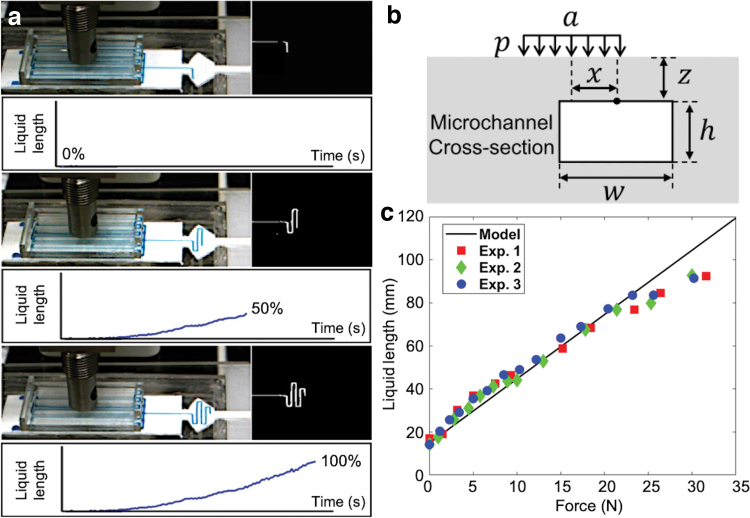
**(a)** Smart Skin sensor calibration process: Fluid in the original position (*top*), compression with increase of the fluid length in the display area (*middle*), and reaching the final position (*bottom*). **(b)** Two-dimensional sketch for defining parameters for analytical modeling. Uniform pressure *p* with width a is applied to *top* surface of elastomer; *w* and *h* are width and height of microchannel, respectively. *x* and *z* define horizontal and vertical locations of microchannel, respectively. **(c)** Experimental data of force response test of Smart Skin with theoretical prediction. Color images are available online.

Force response of the Smart Skin was characterized by applying static forces to a circular area (diameter: 32 mm) on the skin cover, as shown in Figure 5a. Force was increased up to ∼30 N, and an interval of 4 s was given after each increment so that the colored liquid could reach its steady state for each force level. The liquid length was captured and processed in real time using computer vision. Figure 5a shows the snap shots of the experiment and the corresponding vision images and estimated liquid lengths.

For validation, an analytical model was developed to predict the length change of the liquid with external forces. Our model simply calculates the volume change of the moved liquid. When an external force is applied, the microchannels deform with decrease of their volumes. Then, the same amount of the liquid is pushed up to the display area. Our analytical model is based on the three assumptions. First, the colored liquid is incompressible. Second, the air pressure in the empty display channel is negligible compared to the liquid pressure. Finally, the elastic modulus is constant. Based on the linear elastic fracture mechanics, an average of vertical stresses applied to the surface of a crack will decrease the gap between the top and the bottom surfaces by an amount of $\Delta h=2\left(1-\nu^{2}\right)w\sigma_{z}∕E$, where ν and *E* are the Poisson's ratio and the elastic modulus of the material, respectively.^32,65,66^ To correct the contact pressure with the position of the applied force, *p* needs to be substituted with χp, where χ is the correction factor. The change of the area in two-dimensional space can be expressed as:

$$A_{deform}=\Delta hw=\frac{2w^{2}\chi p\left(1-\nu^{2}\right)}{E}$$

where χ depends on the relative positionsx and *z* and the dimension *a* of the applied pressure. As shown in Figure 5b,x is the horizontal distance of the center of the uniform pressure from the center of the microchannel, *z* is the distance between the material surface and the top of the microchannel, and *a* is the width of the applied pressure. χ can be calculated as

$$\chi =\frac{c_{1}c_{2}-c_{3}}{c_{4}}$$

where

$$c_{1}=\tan^{-1}\frac{1+2x}{2z}+\tan^{-1}\frac{1-2x}{2z}$$

$$c_{2}=-8x^{2}a^{2}+32x^{2}z^{2}+8z^{2}a^{2}+16x^{4}y^{4}+16z^{4}$$

$$c_{3}=-16zax^{2}+4za^{3}+16z^{3}a$$

$$c_{4}=\pi \left(4x^{2}+4xa+a^{2}+4z^{2}\right)\left(4x^{2}-4xa+a^{2}+4z^{2}\right)$$

The decreased volume of the microchannel due to the collapse is

$$V=A_{deform}L_{contact}=\frac{2w^{2}\chi pL\left(1-\nu^{2}\right)}{E}$$

where $L_{contact}$ is the length of the microchannel covered by the skin cover, and the increased fluid length in the empty display microchannel is

$$L_{increase}=\frac{V}{A_{display}}=\frac{2w^{2}\chi pL\left(1-\nu^{2}\right)}{El^{2}}$$

Then, the final fluid length can be expressed as

$$L_{final}=L_{initial}+L_{increase}$$

The theoretical predictions and the experimental result are provided in Figure 5c, showing a relatively good agreement. The slight nonlinearity result between the applied force and the liquid length may be due to the following reasons. First, our analytical model is based on the linear fracture mechanics, which assumes that the material has a constant elastic modulus and the model ignores the influence of the channel on global stress distribution. However, the hyperelastic material may have a nonlinear relationship between strain and stress, and its elastic modulus cannot be simplified as a constant. Second, our model assumes that the air pressure does not affect the motion of the liquid. However, the reverse speed increases as the air pocket's length decreases, which indicates that the air pressure may affect the rise of the liquid once the liquid fills in more and more in the display area.

To determine the sensing range of the grip force of our device, we refer to the gripping force study of the hemiplegia stroke patients,^67^ which shows that such patients usually have a grip force <30 N. We also compared the grip force output between different existing medical devices for hand rehabilitation.^61,62^ The force output for those devices are usually <30 N. Therefore, the force range for our system was determined between 0 and 30 N.

### Response time

**FIG. 6.**
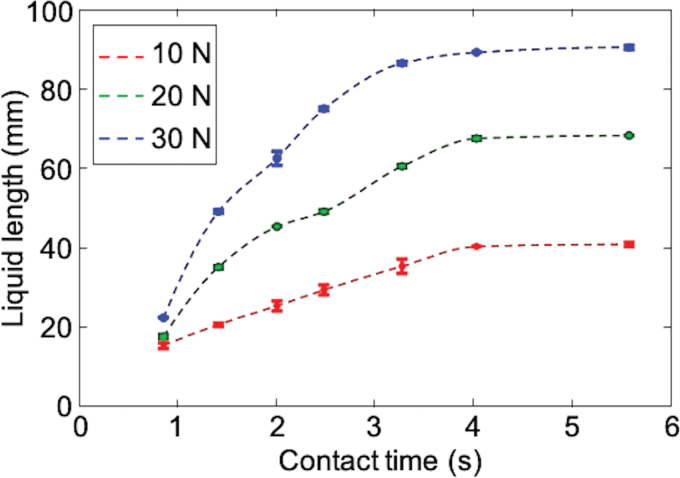
Experimental data of response times with different force levels. Color images are available online.

Response time is an important factor for evaluating the performance of the Soft Skin as a sensor. Experiments were conducted to record the lengths that the colored liquid was able to reach for different levels of force and contact time (i.e., pressure duration). Forces of 10, 20, and 30 N, normal to the skin surface, were applied through the skin cover using the same experimental setup used for the force response test (Fig. 5). For each force level, the contact time was increased, and the maximum liquid length was recorded by computer vision. Figure 6 shows the results of three tests for each force level. For the same contact time, the larger the force was applied, the farther the liquid was able to reach as expected. The liquid length was also proportional to the contact duration. However, the liquid reached steady-state lengths after 4 s regardless of the level of the force.

### Reverse flow speed

While the force response and the response time are directly related to application of force, the characterization of reverse flow of the colored liquid provides information on the sensor performance with the removal of the applied force to the skin. Although fast reverse flow is desirable for short response time, too fast reverse flow easily breaks the liquid and tends to leave residues on the wall surfaces of the microchannel. This is due to the friction and adhesion between the liquid and the microporous structure of the polymer. Since the flow rate is related to the air pressure in the other side of the microchannel, the top air pocket, shown in Figure 2a-ii, needs to be designed to minimize both the liquid residues and the response time at the same time.

**FIG. 7.**
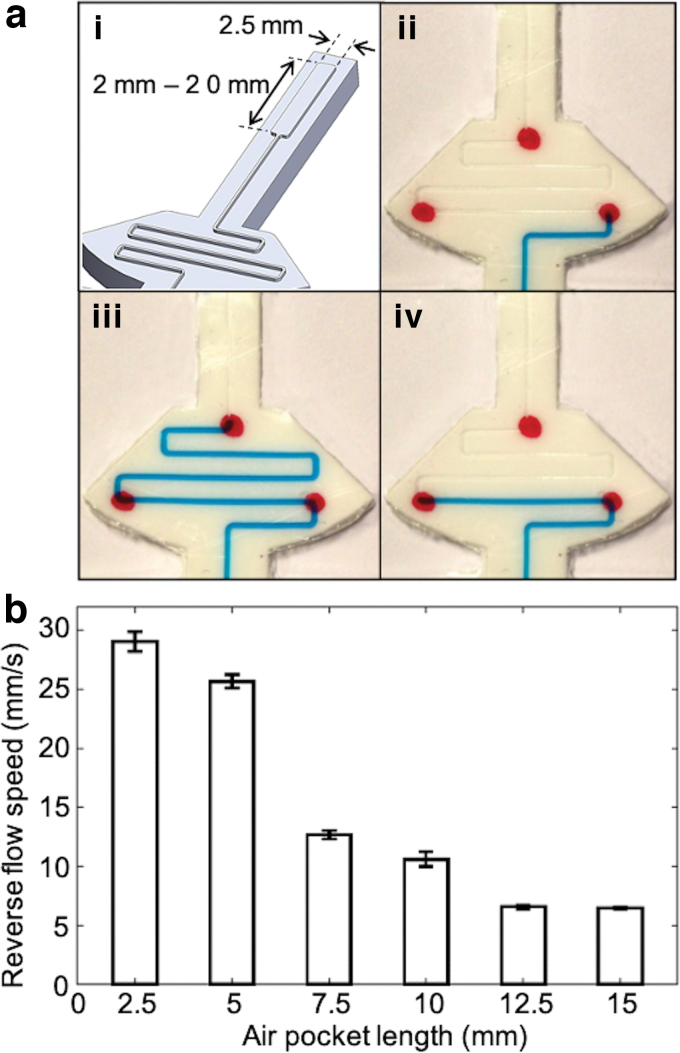
**(a)** Experimental setup for reverse flow test: (i) Varied dimensions of *top* air pocket. (ii) Liquid in the initial position. (iii) Liquid at the highest position with compression of skin. (iv) Liquid at the final position after flowing back with release of compression force. **(b)** Experimental data of reverse flow speeds for different air pocket sizes. Color images are available online.

An experiment was designed to characterize the relationship between the pocket length and the speed of reverse flow. The width of the air pocket was fixed (2.5 mm), but the length was varied from 2.5 to 20 mm with an interval of 2.5 mm (Fig. 7a-i). Three red dots were marked on the skin to show the initial position, the highest position, and the final position of the liquid in the captured images during testing. Starting from the base point (Fig. 7a-ii), the liquid was pushed up to the top point passing the middle point (Fig. 7a-iii). With release of the applied force, the liquid flowed back to the bottom point (Fig. 7-iv). The movement of the liquid from the middle point to the bottom point was recorded and processed by computer vision. The speed of the reverse flow then can be calculated based on displacement and time.

Three identical tests were conducted for each pocket length, and the result is shown in Figure 7b. The speed decreased as the length of the pocket increased reaching a steady state. An air pocket of 10 mm was selected for our prototype based on both the relatively high flow rate and the small number of residues in the microchannel.

## Vision System

In our system, a computer vision approach is used for detecting the grip force on the Smart Skin object. A primary goal of this research is to help reduce the complexity of the rehabilitation system to better support in-home use. By removing the use of embedded electronics in the objects themselves and implementing a passive computer vision capture system instead, we greatly reduce the amount of system maintenance, which also reduces pressure on the stroke patient when setting up and using the system. At the same time, we can also expect consistent levels of resolution and accuracy, even if objects need to be replaced or changed over time.

### Vision accuracy

**FIG. 8.**
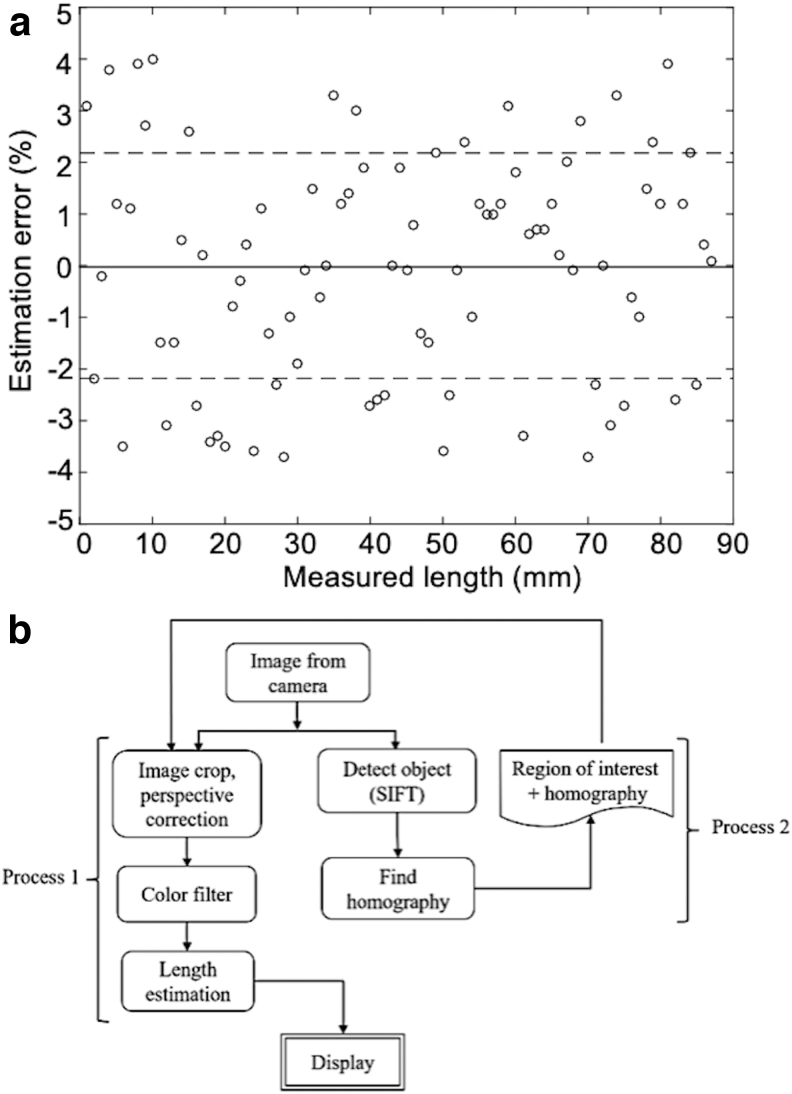
**(a)** Vision error plot for estimating colored liquid length in motion (*circle*: experimental data, *solid line*: mean error, and *dashed line*: RMS of errors). **(b)** Flow chart of image-processing algorithm utilizing SIFT. RMS, root-mean-square; SIFT, scale-invariant feature transform.

The accuracy of the vision system was evaluated using a simple camera system. A video of the Smart Skin with the colored liquid was recorded, and the video frames with increase of the liquid length every millimeter were extracted. The extracted frames were then processed by an image processing algorithm. Finally, the liquid lengths were both manually measured and estimated with computer vision as the length increased. The result is shown in Figure 8a, in which the circles are errors of the estimated liquid length from the actual lengths, and the solid and dashed lines are the mean and the root-mean-square (RMS) values of the vision errors, respectively.

### Image processing

A sample image of the Smart Skin object is taken and fed to the scale-invariant feature transform (SIFT) algorithm,^68,69^ and then, the unique features of the object are saved. These features are used to compare frames to find the object in each image. Once the object is identified, the image is cropped to the size of the object for reduced processing time for the rest of the program.

The next step is to find the homography matrix for perspective correction^70^ using an object detection algorithm. After perspective correction, the color of the corrected image is filtered to obtain the binary image of the liquid channels. Since we do not expect high levels of photometric noise in controlled settings in most homes, we used simple thresholding to detect the liquid region. The liquid was dyed in blue for easy filtering, because its contrast value is significantly high compared to other basic colors, such as red and green. Then, the Canny edge detection algorithm^71^ is implemented to the filtered binary image to convert the image to edges.

Finally, the extremities are identified and converted to a percentage value of the liquid length. Details on the image processing algorithm of the Smart Skin system are depicted in the flowchart in Figure 8b.

The time necessary to process the images is one of the critical factors that determine the bandwidth of the system. In our testing, we were able to process around 35 measurements per second consistently (i.e., ∼28 ms per measurement) using a computer with a 2.9 GHz Intel i7 processor and 16 GB RAM.

### Disturbance rejection

Use of the Smart Skin object often involves different hand motions that may deteriorate the accuracy of the liquid length estimation or cause confusion between channels. To address this issue, we added a cap with special markers on the top area of the object to easily identify each channel and its length. The markers with high contrast and sharp corners provide a clear reference even though the object is in motion.

**FIG. 9.**
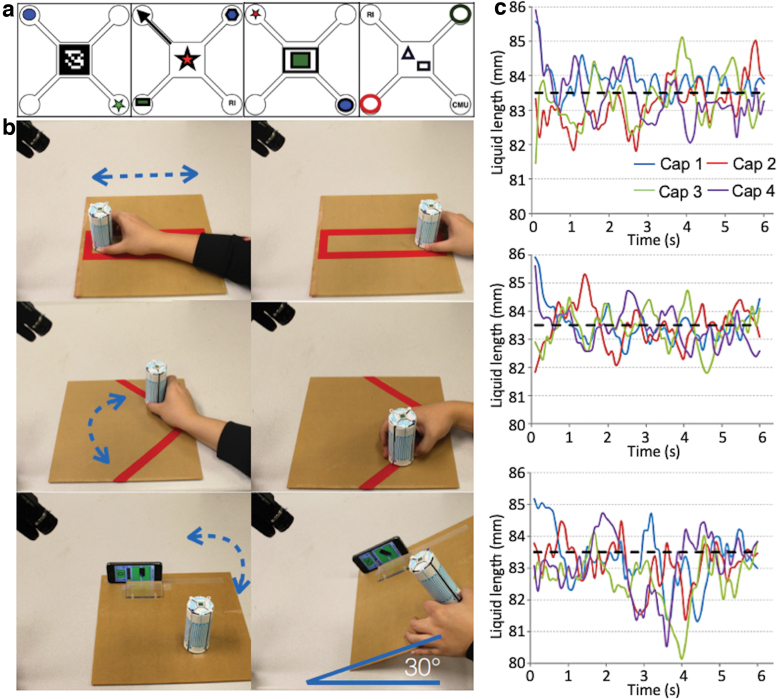
**(a)** Four marker designs for vision processing (Cap 1, Cap 2, Cap 3, and Cap 4 from *left* to *right*). **(b)** Disturbance rejection tests with three different motions: Translation—20 cm (*top*), Rotation—90° (*middle*), and Pitching—30° (*bottom*). **(c)** Length estimation errors from the three disturbance rejection tests: translation (*top*), rotation (*middle*), and pitching (*bottom*). The reference length is shown with a *black dotted line* in each plot. Color images are available online.

Four different caps (Fig. 9a) were prepared and tested with three common hand motions: translation, rotation, and pitching (Fig. 9b). For testing, a known length of one liquid channel was detected with the vision system for the four caps. Figure 9c shows the experimental result of estimating the liquid length with the four caps for the three hand motions. Table 1 summarizes the RMS values of the errors of the estimated lengths with different caps and motions. Among the four cap designs, Cap 1 showed the best overall performance for rejecting different disturbances.

**Table 1.** Root-Mean-Square Values of Length Estimation Errors (Unit: mm)

	Translation	Rotation	Pitching
Cap 1	0.33	0.38	0.84
Cap 2	0.54	0.49	0.62
Cap 3	0.51	0.41	1.37
Cap 4	0.53	0.42	0.97

## System Integration

**FIG. 10.**
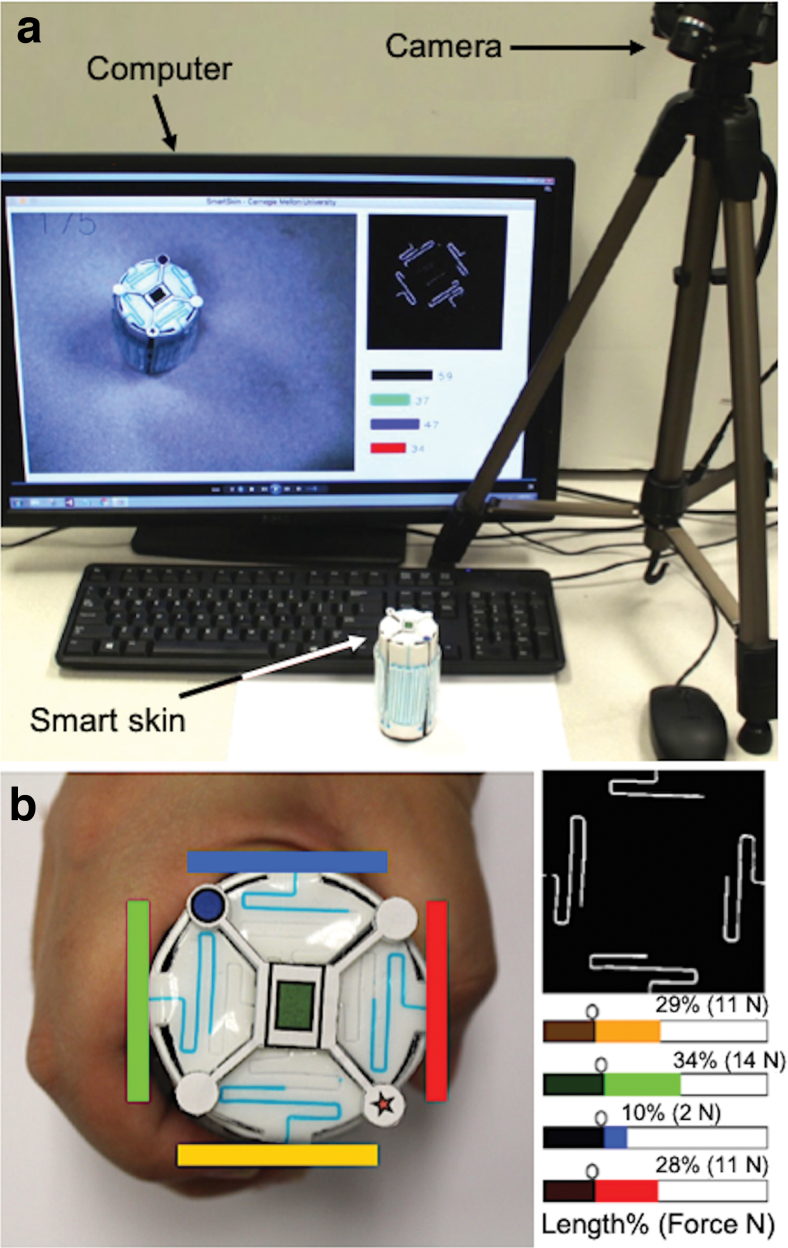
**(a)** Integrated Smart Skin hand rehabilitation system showing major components (*top*) and **(b)** graphical user interface program for Smart Skin hand rehabilitation system (*bottom*). Color images are available online.

Finally, the Smart Skin system for hand rehabilitation was integrated with a regular red–green–blue (RGB) camera and a computer, as shown in Figure 10a. When the patient grasps and releases the Smart Skin object, the camera automatically takes images, and the display shows locations and magnitudes of the forces applied to the object through the graphical user interface program (Fig. 10b) we developed. In the screen, the four liquid channels are identified with four different color bars in the main window (Fig. 10b, left). In the subwindows, real-time filtered image of the object (Fig. 10b, upper and right) and the estimated liquid lengths and their corresponding forces are displayed (Fig. 10b, lower and right). All the force data are saved for further clinical evaluation.

## System Function

Our system aims to help hand stroke patients in their daily rehabilitation exercise and in analyzing their gripping performance. Once the system is set up in a specific location in the patient's living place with a good lighting condition, the patient can either go to the system to do rehabilitation training at a certain period of the day and record the videos or schedule an online meeting with the physical therapist to receive online guidance for training.

During the rehabilitation exercise, the hand/wrist motion can be captured by the camera system. Due to the relative low response time of our sensor, the system may be suitable for assessment of long-period gripping sustainability,^72,73^ in which the patient grips the object with a certain gripping force, lifts it up, and holds it for a long time. In addition, a relative slow gripping force tracking testing,^57,74^ which lets the patient track a slowly changed sinusoidal or square wave, can also be implemented using our system. Our smart skin device is not suitable for repeated gripping tasks with high frequency, which is caused by the limitation of the soft material.

## Discussion

The main contribution of this work is the development of a passive soft sensing force mechanism and its integration with computer vision. The proposed system provides a viable method for in-home hand rehabilitation for stroke patients. The electrical passivity of the training device—the Smart Skin object—eliminates a significant portion of potential maintenance issues for the user, such as having to change or recharge batteries or troubleshoot electrical faults. In addition, the simple computer vision system enables not only real-time monitoring of the user's performance but also automatic recording of the user's activities for regular or future clinical assessments. Our goal is to improve the reliability of the device and the vision algorithm so that the system can become more robust. This will help open up a new space for future rehabilitation technologies that can be implemented and evaluated outside the clinic.

In this study, although we proved the viability of the soft sensing technology and demonstrated its feasibility through the Smart Skin system, there are several limitations to be addressed for the proposed system to be more effective and practical.

When the Smart Skin is used multiple times, the colored liquid sometimes leaves residues on the wall of the microchannels. Although we tried to reduce this effect by coating the channel wall with a lubricant, the lubricant did not stay on the wall permanently. To address this issue, we are currently investigating different surface treatment techniques to make the channel walls hydrophobic so that the liquid does not break when it retreats, as discussed in Epstein *et al*.^75,^ and Kim *et al*.^76^

The current Smart Skin uses compliant silicone as a base material and embedded microchannels with colored liquid as a method for visual display. Although it worked well during our experiments in a controlled environment, the level of the colored liquid in the microchannels may vary with environmental conditions, such as changes of ambient temperatures and pressure, due to the contraction or expansion of the captured air in the channels. These changes may cause different initial levels of the colored liquid, requiring a recalibration process every time the system starts. Investigation of more stable materials than air is an area of our future work.

Another area of future work is investigation of different object designs. Depending on various physical features, such as shape, size, weight, curvatures, textures, and hardness, the rehabilitation progress or performance of the stroke patients may be different. Once the optimum features are decided, we can customize the design of the soft sensors and the vision algorithms.

In addition, we assumed a relatively low photometric noise level in image processing, since the system would be used always indoors where the ambient light can be easily controlled. Even though the lighting condition changes, the Smart Skin uses only a single color for the liquid with a white background that can be easily recognized. In other words, the colors of the liquid and the display area will have different color gradients even under different lighting conditions. Furthermore, we can crop the image to only contain the object, since we know what kind of pattern we are looking for in the image. However, characterization of the photometric noises and their influence to the performance of the system will be an area of future work.

Finally, clinical testing of the Smart Skin system with stroke survivors should be conducted following the design and development stage. The clinical test will provide information on the efficacy and impact of the approach and also allow for further improvement of the proposed system.

## Conclusion

The Smart Skin in-home rehabilitation system was developed utilizing a microfluidic color-changing soft material combined with a simple computer vision system. The Smart Skin comprises a compliant silicon rubber layer that contains embedded microchannels filled with a colored liquid. When the skin is compressed, the liquid in the microchannel moves to a different place and the advancement of the liquid is detected by an RGB camera. Experimental results showed an estimation of a gripping force up to 25 N with an error of <4%.

A manufacturing method using layered molding and casting was developed to build the Smart Skin. The internal walls of the microchannels were coated with a lubricant to minimize the residue of the colored liquid when the liquid retraced. The liquid in the current prototype required about 4 s to reach its initial position when the contact pressure was removed.

A computer vision system using the SIFT algorithm was implemented to detect the color change of the microchannels. The system showed an ability to reject disturbances from different hand motions using special markers on the top area of the Smart Skin object. Finally, a user-interface program was developed to display the gripping forces and their locations in real time.

## Supplementary Material

Supplemental data

Supplemental data
