# Supplementary material for: Smart Skin: Vision-Based Soft Pressure Sensing System for In-Home Hand Rehabilitation
Source: Soft Robot. 2022 Jun 8;9(3):473–85. doi: 10.1089/soro.2020.0083 (PMC9232239; doi:10.1089/soro.2020.0083)
Supplement: Supplemental data [file Supp_DataS1.pdf]

Smart Skin: Vision-Based Soft Pressure Sensing System for In-Home Hand Rehabilitation

Yuanfeng Han, Aadith Varadarajan, Taekyung Kim, Gang Zheng, Kris Kitani, Aisling Kelliher, Thanassis Rikakis, and Yong-Lae Park

Supplementary Document

Influence of Contact Area

In addition to the microchannel geometries, the way of compression may generate different output levels of the liquid. After covering the liquid chamber with a rigid cover, we changed the location of compression and measured the sensitivity. The sensitivity was highest when the middle part was compressed, but compression of the edge decreased the sensitivity because it tilted the cover and did not effectively distribute the force (Fig. S1).

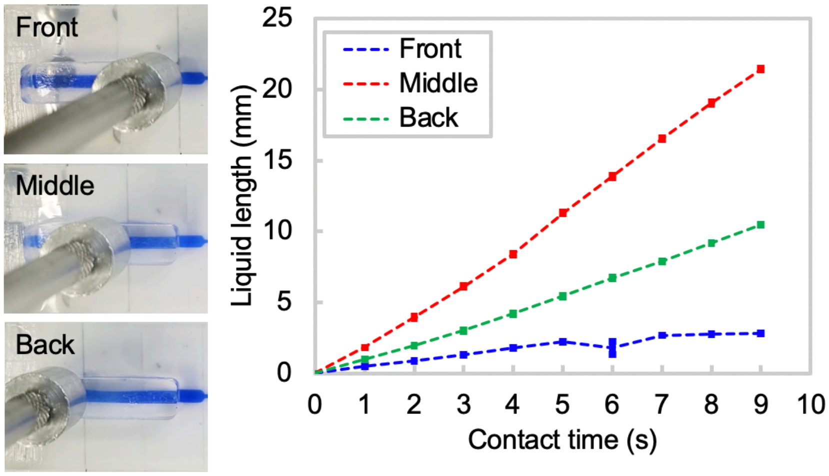

Figure S1: Influence of contact area to the sensitivity of the movement of liquid. The sensitivity was highest when the middle area was compressed.

Cyclic Test

A cyclic test was also conducted to check the reliability and the durability of the skin. The skin was compressed and released 500 times. Although the skin showed physical robustness in this test, the end position of the liquid gradually increased (Fig. S2). This is due to the leakage of the air in the pocket through the micropores of the silicone material. Decreasing the air permeability of the silicone material will be one of the immediate areas of future work.

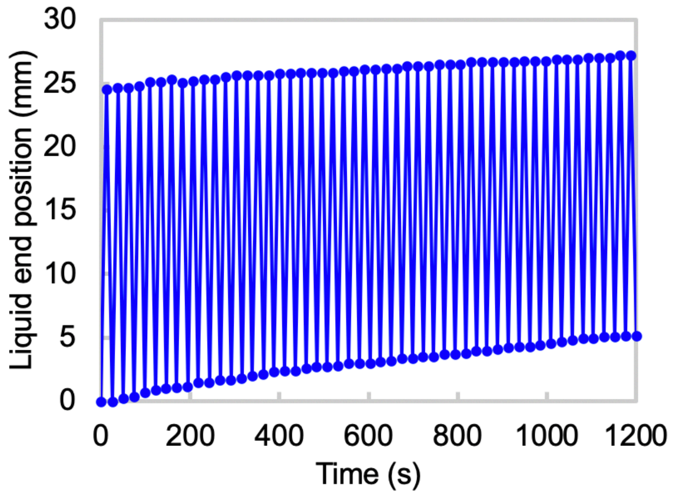

Figure S2: Result of cyclic test for a single channel soft skin.

Skin Material Characterization

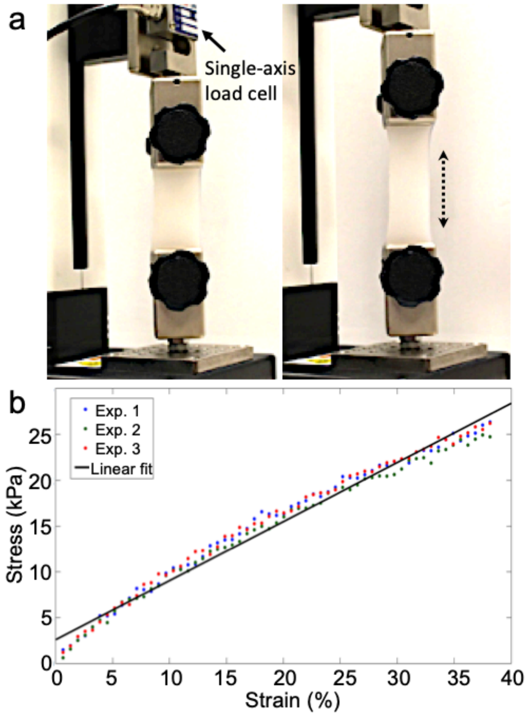

Figure S3: Stress-strain test: (**a)** Skin sample before (left) and after stretched (right). (**b)** Result from three identical experiments and linear fit to the estimated *E*. The experimental elastic modulus of the skin material was 64.4 kPa.
